# Genetic and phenotype recovery of *Ananas comosus* var. MD2 in response to ionizing radiation

**DOI:** 10.1038/s41598-022-26745-3

**Published:** 2023-01-05

**Authors:** Siyuan Ma, Anis Norsyahira Mohd Raffi, Muhamad Afiq Rosli, Nurul Amalina Mohd Zain, Mohd Hafiz Ibrahim, Saiful Anuar Karsani, Jamilah Syafawati Yaacob

**Affiliations:** 1 Institute of Biological Sciences, Faculty of Science, Universiti Malaya, 50603 Kuala Lumpur, Malaysia; 2 Department of Biology, Faculty of Science, Universiti Putra Malaysia, 43400 Serdang, Malaysia; 3 Centre for Research in Biotechnology for Agriculture (CEBAR), Universiti Malaya, 50603 Kuala Lumpur, Malaysia

**Keywords:** Biotechnology, Plant physiology

## Abstract

Due to their sessile nature, plants are exposed to various environmental stressors such as exposure to high levels of harmful ultraviolet (UV), ionizing, and non-ionizing radiations. This exposure may result in various damages, ranging from DNA and chromosomal aberrations to phenotypic abnormalities. As an adaptation, plants have evolved efficient DNA repair mechanisms to detect and repair any damage caused by exposure to these harmful stressors to ensure their survival. In this study, the effects of gamma radiation (as a source of ionizing radiation) on clonal *Ananas comosus* var. MD2 was evaluated. The morphology and physiology of the clonal plantlets before and after exposure to gamma radiation were monitored at specific time intervals. The degree of genetic variation between the samples pre- and post-irradiation was also analyzed by using inter-simple sequence repeat (ISSR) markers. The resulting data revealed that the heights of the irradiated plantlets were significantly reduced (compared to control), but improved with the recovery period. Irradiated samples also exhibited relatively good photosynthetic efficiency that further improved as the plantlets recover. These observations were supported by the ISSR analysis, where the genetic dissimilarities between the irradiated samples and control were reduced by 0.1017, after 4 weeks of recovery. Overall, our findings suggested that the phenotype recovery of the clonal *A. comosus* var. MD2 plantlets was contributed by their ability to detect and repair the DNA lesions (as exemplified by the reduction in genetic dissimilarity after 4 weeks) and hence allow the plantlets to undergo phenotype reversion to normal plant stature.

## Introduction

Pineapple *(Ananas comosus),* a member of the bromeliad family is the third commercially important tropical fruit crop in the world^1^. Over the past few decades, pineapple has been commercially used for many purposes, either for fresh consumption or as canned food^1^. The MD2 variety of pineapple is prominent in the global market due to superior characteristics such as better colour, flavour, good sweetness to acidity balance, juiciness, and longer shelf life^2^. In Malaysia, various cultivars of pineapple are planted, including Moris, Gajah, Gandul, Yankee, Sarawak, N36, Josapine, and MD2. As the market demand for MD2 is higher compared to other pineapple cultivars, it has been identified as a key crop under Malaysia’s National Key Economic Area (NKEA) of the Economic Transformation Program (ETP)^1^. Unfortunately, the MD2 variety is susceptible to serious pineapple diseases such as fungal black rot (caused by *Thielavipos paradoxa*) and bacterial heart rot (caused by *Erwinia chrysanthemi*)^1^.

Due to their sessile nature, plants are exposed to a variety of stressors that arise from their surroundings such as abiotic and biotic stresses. Abiotic stresses are physical stresses such as drought, salinity, extreme temperature, chemical toxicity, and oxidative stress. They can lead to morphological, physiological, biochemical, and molecular changes that will critically affect plant growth and productivity^3^. These changes may lead to the denaturation of functional and structural proteins which in turn affect the functionality of plant growth mechanisms. Apart from that, other abiotic stress such as exposure to radiation can induce cellular and molecular damages, particularly in DNA structure^4^.

Ionizing radiation induced by gamma radiation produces a large number of lesions in plants. This is affected through direct and indirect interactions with DNA molecules via water radiolysis, which generates highly reactive species such as hydroxyl (OH) radicals, free electrons, and hydrogen radicals^5^.·OH radicals are the most reactive of all ROS (reactive oxygen species). It is able to reach various biomolecules such as DNA, lipids, proteins, and almost any constituent of the cell^5^. The most commonly encountered DNA damage are changes in structure and function that include modification of nucleotides and breaks of the phosphodiester bonds^6^. The type of damage induced by gamma irradiation depend on the dosage of exposure. Studies on γ-rays by Kovalchuk, et al.^7^ on *Populus nigra* var. *italica* suggested that the regulation of repair mechanisms is necessary for adaptive responses towards ionizing radiation. Nevertheless, the information on DNA damage accumulation and molecular mechanisms pertaining to radiation injury at different dosage is still limited^4^. According to Ros and Tevini^8^, the most frequent damage inflicted by radiation exposure is hormonal imbalance, caused by the interference in indoleacetic acid (IAA) metabolism. Radiation-induced damage may affect the plant’s DNA, which in turn results in heritable mutations, if not repaired before replication or any physiological process^9^. Thus, mechanisms to repair these mutations are required to maintain the necessary genetic integrity.

In this study, the effect of gamma radiation on clonal *Ananas comosus* var. MD2 was evaluated. The morphological and physiological characteristics of the clonal plantlets before and after exposure to gamma radiation were monitored at different time intervals (at different post-recovery periods). Additionally, the degree of genetic variation between the samples pre- and post-irradiation was also analyzed using inter-simple sequence repeat (ISSR) markers. This study will provide an understanding on how plants repair the damage induced through exposure to ionizing radiation, and understand the mechanism of adaptive plasticity employed in repairing its phenotype post-irradiation.

## Materials and methods

### Plant materials and gamma irradiation

Two-month-old in vitro grown *Ananas comosus* var. MD2 plantlets were used as the starting material for this study. Leaf base explants were harvested and used to induce direct regeneration of this species. The explants were cultured on Murashige and Skoog (MS) media supplemented with 1.0 mg/L IBA (Indole-3-butyric acid) and 2.0 mg/L BAP (6-Benzylaminopurine)^10^. Cultures were maintained at 25 ± 1 °C with a photoperiod of 16 h light and 8 h dark, under light illumination of 1000 lux. In this experiment, all explants were harvested and cultured from the same mother plant to produce genetically identical (clonal) plantlets.

The clonal plantlets were irradiated with gamma radiation (Gamma Cell 220, Canada) at the Physics Laboratory, Department of Physics, Faculty of Science, Universiti Malaya, Malaysia. In total, 30 plantlets were irradiated at a previously determined optimum radiation dosage of 400 Gy. Non-irradiated plantlets (30 plantlets) were used as control. The optimum radiation dosage was determined based on the LD_50_ (lethal dose for 50% of the plants tested, or radiation intensity that caused a 50% mortality rate). Following irradiation, the plantlets were kept in the culture room to allow recovery.

### Morphological observation and physiological analysis

The morphology of the plantlets was observed and recorded at various recovery periods; day 1, week 1, week 2, week 4 and week 8 after irradiation. Morphological characteristics such as height, leaf width (based on the D-leaf), and occurrences of chlorosis and leaf variegation were observed and recorded. Concurrently, the physiology of the plantlets was also observed and recorded. The physiological parameters recorded were chlorophyll content, net photosynthesis rate, stomatal conductance, transpiration rate and water use efficiency (WUE).

#### Chlorophyll content

For the measurements of leaf chlorophyll content, the *in-situ* chlorophyll content (SPAD) was determined using the SPAD-502 meter (Konica Minolta Optic Inc., Tokyo, Japan). All data were recorded at every post-recovery period, in triplicates.

#### Leaf gas exchange

The net photosynthesis rate, stomatal conductance, transpiration rate, and water use efficiency (WUE) of the samples were evaluated using a closed infra-red gas analyzer LICOR 6400 Portable Photosynthesis System (IRGA, Licor Inc., Lincoln, NE, USA).

### DNA extraction and quantification

Total DNA was extracted from leaf materials obtained from 10 regenerated clonal plantlets (randomly collected), pre- and post-irradiation. This was done for each recovery period (day 1, week 1, week 2, week 4 and week 8). Briefly, 100 mg (0.1 g) of plant leaves were weighed and subjected to DNA extraction. DNA was purified from the fresh leaves using the Gene Matrix Plant and Fungi DNA Purification Kit (EURx Ltd., Poland) according to the manufacturer’s instructions. The concentration of extracted DNA was determined using a Gen 5.0 Microplate Reader and Image Software, BioTek Gen 5 (BioTek Instrument, USA). The DNA samples were then stored at − 80 °C in an Ultra-Low Temperature Freezer (New Brunswick, USA) until use.

### ISSR analysis

A total of 20 ISSR primers were tested (data not shown). Only 8 of the 20 primers yielded clear and scorable bands with acceptable intensity. Thus, these primers (Eurx Molecular Biology Product, EURx Ltd.) were used in the analysis using a Polymerase Chain Reaction (PCR) method. The sequences of the 8 ISSR primers used are shown in Supplementary Table S1^11^, with Table 1 listing the PCR components used in the study. Amplifications were performed in a Mastercycler Nexus Thermal Cycler (Eppendorf, North America). The PCR was carried out at pre-determined optimum annealing temperatures for each primer. The ISSR PCR reactions were performed with an initial denaturation process at 94 °C for 5 min, followed by 35 cycles of 1 min at 94 °C for denaturation, 1 min annealing, and 2 min at 72 °C for the extension. The PCR reaction was ended with 1 final extension step for 10 min at 72 °C. Resulting products were cooled at 10 °C. For analysis, the PCR products were subjected to an electrophoretic separation using EPS-300X (C.B.S. Scientific Company, United State). A total of 8 μl of amplified products was separated on 1.5% Agarose Electrophoresis Grade (EURx Ltd., Poland) in 1 × TAE buffer for about 90 min at 50 V. Gels were stained using Red Safe Nucleic Acid Staining Solution (Intro Biotechnology, Korea). The gels were then visualized under UV light using the AlphaImager™ Gel Imaging System (Alpha Innotech, Germany).

**Table 1:** PCR components in each total reaction volume.

Components	Concentration	Volume added
Template DNA	50 ng µl^−1^	x µl
Primer (Integrated DNA Technologies, USA)	2 µM	1 µl
Buffer C (Eurx Ltd.)	10 x	2 µl
MgCl_2_ (Eurx Ltd.)	25 mM	2 µl
BSA (Invitrogen, USA)	4 mg/μL	1 µl
Taq DNA Polymerase (Eurx Ltd.)	1.25 U/µl	1 µl
dNTPs (Eurx Ltd.)	4 mM	1 µl
ddH_2_O	–	x µl
Total reaction volume		20 µl

### Band scoring and Jaccard analysis

The banding profiles generated by all the ISSR primers were analyzed and scored to calculate genetic dissimilarities between samples. This allowed us to determine the genetic variation that resulted due to gamma radiation. The bands were scored 1 as the presence of a band and 0 as the absence of a band for each irradiated and control plant sample. The scored bands were then transformed into a binary character matrix to calculate the Jaccard’s distances, to determine the genetic dissimilarity and variation between the clonal plantlets of *Ananas comosus* var. MD2 (irradiated versus non-irradiated) at each recovery period. For this purpose, the DARwin 6.0 software was used.

### Statistical analysis

Data were analyzed either using Student’s t-test or ANOVA in SPSS (Statistical Package for the Social Sciences, Version 25) with Duncan’s multiple range tests (DMRT) as the post hoc test at p < 0.05.

### Ethics approval and consent to participate

All procedures were conducted in accordance to the guidelines.

## Results

### Production of clonal plantlets and assessment of genetic similarity

Following successful generation of clonal *A. comosus* var. MD2 plantlets, the leaves of the plantlets were harvested and used for ISSR analysis, to assess the genetic similarity between the plantlets. The Jaccard’s dissimilarity distance between clonal samples are shown in Table 2. This displays the level of dissimilarity between samples, whereby the genetic distance is provided in decimal forms, ranging between zero (0) indicating no genetic variability, to one (1) indicating highest genetic variability. In this study, the genetic distance between all the clonal samples (Table 2) ranged from 0.0291 to 0.1628, implying that the samples were of clonal origin, and have very low genetic variability.

**Table 2:** The Jaccard’s dissimilarity distances generated from 10 randomly selected clonal plantlets**.**

	Plantlet A	Plantlet B	Plantlet C	Plantlet D	Plantlet E	Plantlet F	Plantlet G	Plantlet H	Plantlet I
Plantlet B	0.076392								
Plantlet C	0.076392	0.029143							
Plantlet D	0.076392	0.076392	0.076392						
Plantlet E	0.076392	0.076392	0.076392	0.076392					
Plantlet F	0.076392	0.076392	0.076392	0.076392	0.076392				
Plantlet G	0.076392	0.076392	0.076392	0.076392	0.076392	0.076392			
Plantlet H	0.162808	0.162808	0.162808	0.162808	0.162808	0.162808	0.162808		
Plantlet I	0.162808	0.162808	0.162808	0.162808	0.162808	0.162808	0.162808	0.060896	
Plantlet J	0.076392	0.076392	0.076392	0.076392	0.076392	0.076392	0.076392	0.162808	0.162808

The Jaccard’s dissimilarity distances were then used to compute a phylogenetic tree using DARwin 6.0 software. For this purpose, a cluster analysis was performed based on dissimilarity coefficients generated from the ISSR data of the 62 scorable bands (generated using the 10 randomly selected clonal plantlets). The results were then used to generate a UPGMA dendrogram (Supplementary Fig. S1). The dendrogram showed that 8 out of the 10 clonal plantlets were grouped in a single cluster, while plantlets H and I were grouped in a separate single cluster. However, they demonstrated a very low rate of polymorphism (with only 0.16281 dissimilarity indices). This suggested H and I exhibited some degree of variation, but with very low Jaccard’s distance compared to the other plantlets.

### Morphology of plantlets following gamma irradiation

Following exposure to gamma radiation, all samples (irradiated and non-irradiated) were monitored for 8 weeks and the morphological characteristics (plant height, leaf width, leaf colour, and leaf variegation) of the plantlets were observed and recorded. Supplementary Fig. S2a shows the morphology of the non-irradiated (control) *A. comosus* plantlet. The control plantlet was shown to have sword-like and bear sharp, up-curved leaves. The colour of the leaves were uniformly green and the leaf surfaces were waxy.

**Figure 1:**
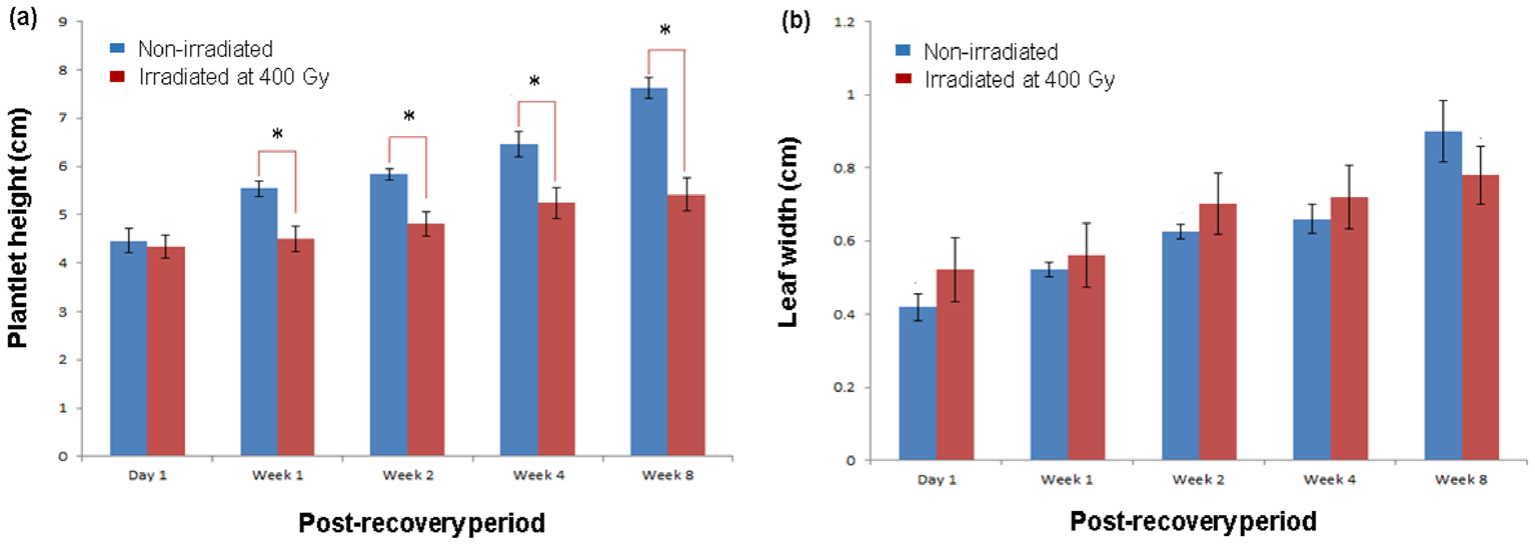
(**a**) Plantlet height, and (**b)** leaf width at five different post-recovery periods after being irradiated with gamma at 400 Gy. *significantly different at p < 0.05.

In comparison to control, plantlets irradiated with gamma at 400 Gy showed a reduction in plantlet height (Fig. 1a). The height of the non-irradiated (control) plantlets continuously increased during the 8 weeks of observation. On the other hand, irradiated plantlets were observed to be stunted and showed significant height differences compared to non-irradiated (control) plantlets after 1, 2, 4 and 8 weeks of post-recovery periods. Nevertheless, the height of the irradiated samples was shown to also increase with the recovery period (although at a much slower rate than control plantlets). This implied that irradiated plantlets had undergone phenotype repair and were slowly recovering after exposure to gamma irradiation. Other than plant height, the width of leaves of irradiated and non-irradiated (control) plantlets were also measured and compared. We found that the leaf width of both irradiated and non-irradiated (control) plantlets were not significantly different (p > 0.05, when observed at all post-recovery periods) and also increased with time (Fig. 1b).

Leaf colour, chlorosis, and any occurrence of leaf variegation on the samples were also monitored for 8 weeks. Supplementary Fig. S2 shows the gamma-irradiated plantlets after 2 weeks of recovery. It was observed that at 2 weeks post-irradiation, some leaves of the gamma-irradiated plantlets started to undergo chlorosis and showed patterns of leaf variegation or differential chlorophyll distribution (Supplementary Fig. S2b,c).

### Physiology of plantlets following gamma irradiation

#### In-situ chlorophyll content (SPAD value)

**Figure 2:**
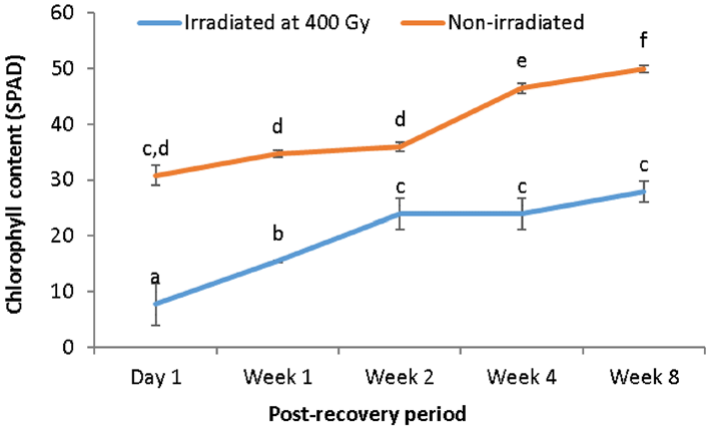
SPAD values of non-irradiated, and irradiated *A. comosus* plantlets at five different post-recovery periods.

In general, the SPAD values of both the non-irradiated (control) and irradiated plantlets were observed to increase with time (Fig. 2). However, the increase was observed to be non-significant in the irradiated plantlets after week 2 of the recovery period. The chlorophyll content of the irradiated plantlets was also observed to be significantly lower than that exhibited by the non-irradiated; control plantlets (Fig. 2) at all time points.

#### Leaf gas exchange

**Figure 3:**
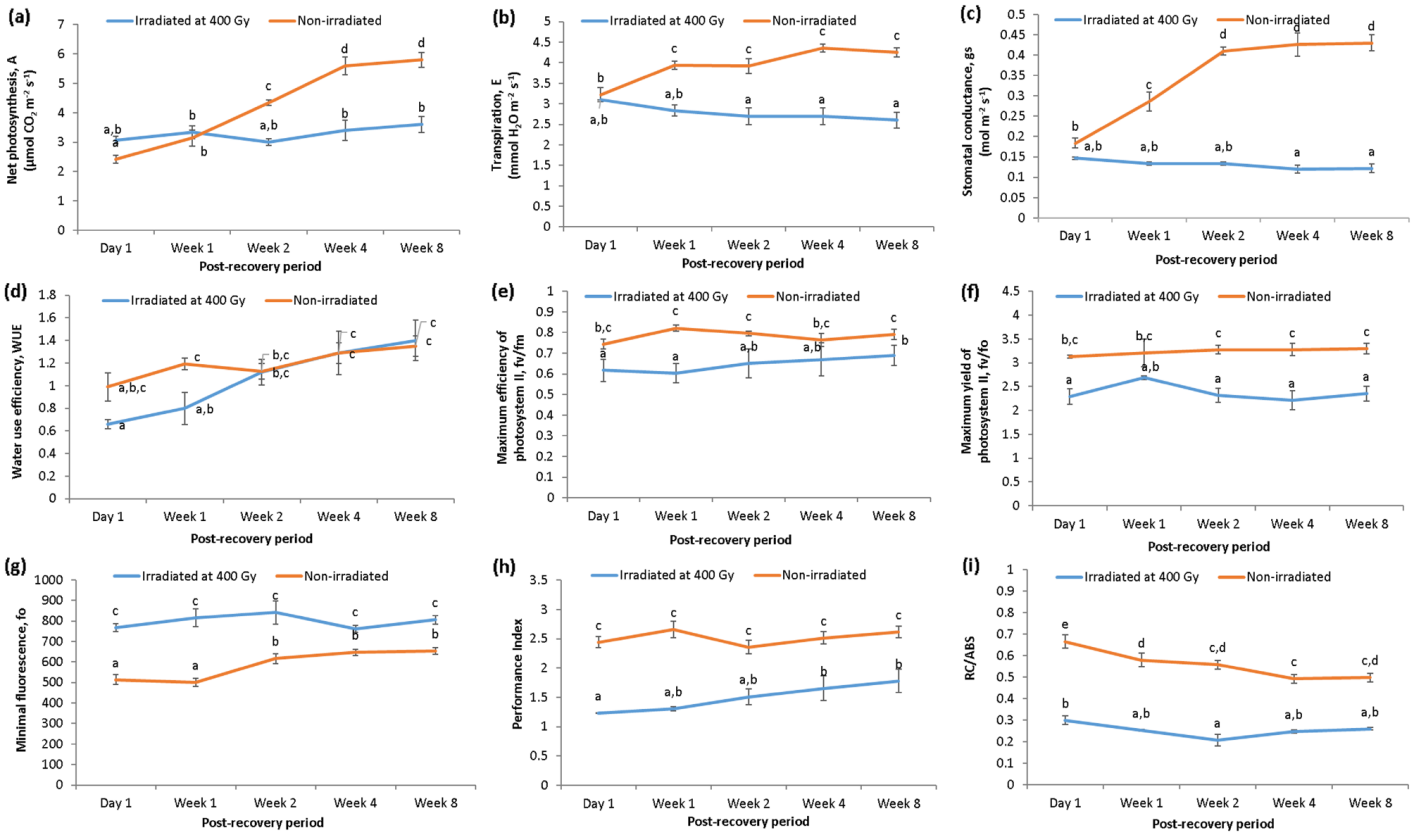
(**a**) Net photosynthesis, (**b**) transpiration rate, (**c**) stomatal conductance, (**d**) water use efficiency, (**e**) maximum efficiency of photosystem II, (**f**) maximum yield of photosystem II, (**g**) minimal fluorescence, (**h**) performance index, and (**i**) RC/ABS (density of reaction centre per PSII antenna) of non-irradiated and irradiated *A. comosus* plantlets at five different post-recovery periods.

The photosynthesis rate (A) of irradiated plantlets was observed to be stable with no significant difference throughout the 8 weeks period. On the other hand, the photosynthesis rate of non-irradiated (control) plantlets was observed to increase with time, and peaked after 4 weeks (Fig. 3a). Similarly, the transpiration rate (E) and stomatal conductance (gs) of irradiated plantlets were also found to be relatively constant and not significantly different throughout the 8 weeks period (Fig. 3b,c). For non-irradiated plantlets, the transpiration rate and stomatal conductance were found to increase with time, similar to the trend shown by the photosynthesis rate. They started to plateau after 1 and 2 weeks, respectively. On the other hand, the water use efficiency (WUE) of the non-irradiated plantlets was higher than irradiated plantlets (day 1 and week 1) and both were shown to increase with time (Fig. 3d). However, the increase was observed to be non-significant after 2 weeks of recovery (Fig. 3d). Of interest, the WUE of both non-irradiated and irradiated plantlets was found to be similar from the post-recovery period of week 2 to week 8.

As shown in Fig. 3e,f, the maximum efficiency (fv/fm) and yield (fv/fo) of photosystem II in control (non-irradiated) plantlets were observed to be significantly higher than that of irradiated plantlets at all time points. The fv/fm and fv/fo values of both the non-irradiated and irradiated plantlets were also shown to be constant and not significantly different throughout the 8 weeks recovery period. In contrast, the minimal fluorescence (fo) of the irradiated plantlets was shown to be higher than that of control (non-irradiated) plantlets at all time points (Fig. 3g). The fo of the irradiated plantlets was observed to be constant throughout the 8 weeks period, while the fo of non-irradiated plantlets was shown to increase at week 2, and plateaued from that point onwards.

The performance index and RC/ABS (density of reaction centers per PSII antenna) of the plantlets were also monitored. It was found that the performance index and RC/ABS values of the irradiated plantlets were observed to be significantly lower than that of control (Fig. 3h,i). As shown by Fig. 3h, the performance index of both irradiated and non-irradiated plantlets exhibited an increasing trend, however, the increase was not statistically significant. In contrast, The RC/ABS values of both irradiated and non-irradiated plantlets were observed to decrease with time (Fig. 3i).

### ISSR analysis following gamma irradiation

**Figure 4:**
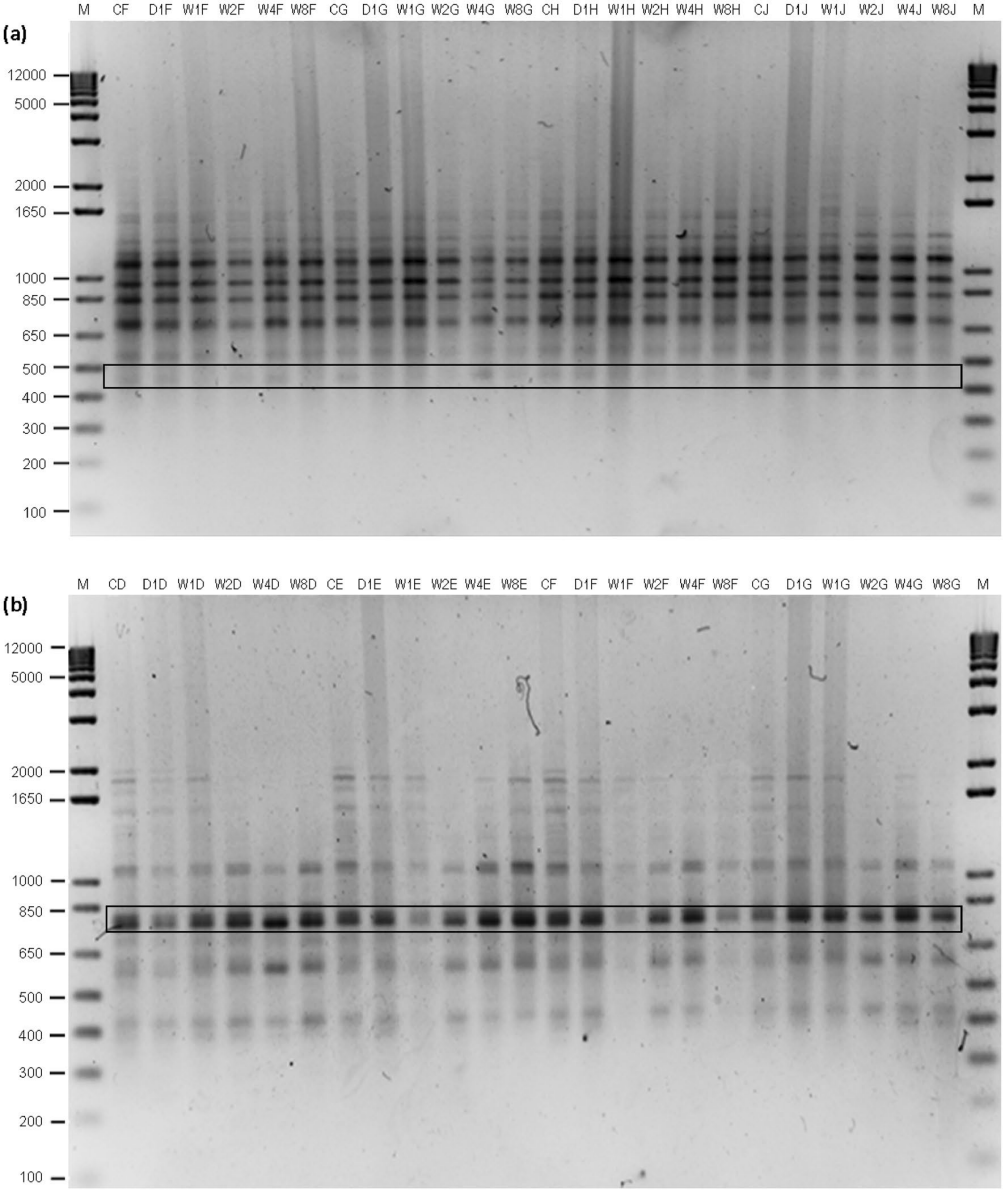
(**a**) Gel image obtained using ISSR primer UBC 840, where the highlighted portion of the image shows the presence of polymorphic bands, and (**b**) gel image obtained using ISSR primer UBC 841, where the highlighted portion of the image shows the presence of monomorphic bands. *C* control plantlet, *A-J* Plantlets A-J, *D1* Day 1, *W1-8* Week 1–8. The original gel is presented in Supplementary Fig. S4, S5.

The leaves of 10 randomly selected gamma-irradiated and non-irradiated (control) plantlets were harvested at each post-recovery period. DNA was extracted and subjected to PCR analysis using 8 ISSR primers, as described in the methods section. The presence of polymorphic bands generated using ISSR primer UBC 840, from 10 randomly selected irradiated and non-irradiated (control) clonal *Ananas comosus* var. MD2 plantlets at various post-recovery periods (day 1 until week 8) is shown in Fig. 4a. A mean number of 12 scorable bands per primer were obtained, and this primer amplified a total of 667 bands from all samples (Table 3). The size of the amplified fragments ranged from 400 to 2000 bp (Table 3). On the other hand, Fig. 4b shows the presence of monomorphic bands generated using ISSR primer UBC 841. This primer generated 12 scorable bands and amplified a total of 653 bands from all samples (Table 3). The size of the amplified fragments ranged from 400 to 2000 bp (Table 3).

**Table 3:** Total, mean number and frequency of monomorphic and polymorphic bands for each ISSR primer from 10 randomly selected gamma-irradiated and non-irradiated plantlets.

Primers code (UBC)	Total number of bands amplified	Mean number of scorable bands per primer	Total number and mean number of polymorphic bands per primer	Total number and mean number of monomorphic bands per primer	Frequency of polymorphic bands	Range of amplification (bp)
UBC_807	249	8	67 (6.7%)	13 (1.3%)	83.75	500–2000
UBC_809	300	8	67 (6.7%)	13 (1.3%)	83.75	500–1650
UBC_829	346	7	42 (4.2%)	28 (2.8%)	60	500–2000
UBC_834	754	13	17 (1.7%)	113 (11.3%)	13.08	400–1650
UBC_840	667	12	23 (2.3%)	97 (9.7%)	19.17	400–2000
UBC_841	653	12	39 (3.9%)	81 (8.1%)	32.5	400–2000
UBC_855	736	13	34 (3.4%)	96 (9.6%)	26.15	650–5000
UBC_856	464	8	14 (1.4%)	66 (6.6%)	17.5	650–1650
Total	4169	81	303 (37.4%)	507 (62.6%)		

Overall, a total of 4169 bands were amplified from the control and gamma-irradiated plantlets (Table 3). The size of the amplified fragments ranged from 400 to 5000 bp. The mean number of scorable bands per primer varied from 7 to 13. Both primers UBC 807 and UBC 809 generated the highest number of polymorphic bands, with a total of 67 polymorphic bands (83.75%) and 13 monomorphic bands. The size of the amplified fragments for primer UBC 807 ranged from 500 to 2000 bp, while for primer UBC 809, they ranged from 500 to 1650 bp (Table 3). In contrast, primer UBC 834 only produced 17 polymorphic bands (13.08%) and 113 monomorphic bands. In this study, it was shown that all 8 ISSR primers were able to detect the occurrence of polymorphism in the samples. Out of 4169 amplified bands produced by all primers, the highest polymorphic bands were produced from primers UBC 807 and UBC 809, where an average of 6.7 out of the 8 scorable bands per primer was polymorphic. Those primers were detected to be the most reproducible, as they resulted in the highest polymorphism among all samples tested.

### Jaccard’s distance analysis and UPGMA dendrogram following gamma irradiation

The binary character matrix generated from gamma-irradiated and non-irradiated (control) samples at various post-recovery periods were also used to calculate the Jaccard’s distances using DARwin 6.0 software. The Jaccard’s dissimilarity distance table between the clonal samples is shown in Table 4. Data analysis revealed that the genetic distance between all samples ranged from 0.0821 to 0.2073. The highest genetic distance (0.2073) was observed between the non-irradiated (control) and gamma-irradiated plantlets after 1 day to 2 weeks post-irradiation. Interestingly, the genetic distance between the control and irradiated plantlets was observed to decrease after 4 weeks of recovery, resulting in a genetic distance of 0.1017.

**Table 4:** The Jaccard’s dissimilarity distances between clonal *Ananas comosus* var. MD2 plantlets after gamma radiation treatment at different post-recovery periods (Day 1, Week 1, Week 2, Week 4 and Week 8), compared to control.

	Control	Day 1	Week 1	Week 2	Week 4
Day 1	0.207276				
Week 1	0.207276	0.207276			
Week 2	0.207276	0.207276	0.207276		
Week 4	0.101671	0.207276	0.207276	0.207276	
Week 8	0.101671	0.207276	0.207276	0.207276	0.082113

The Jaccard’s dissimilarity coefficients were also used to construct a UPGMA dendrogram to show the genetic relationship between the samples at different post-recovery periods. Based on the UPGMA dendrogram shown in Supplementary Fig. S3, the samples were divided into three main clusters, with irradiated plantlets at the post-recovery period of week 4 and week 8 being the closest to the control plantlets. On the other hand, irradiated plantlets from the post-recovery period of day 1 until week 2 were grouped together and were the most distant from the control. This observation further supports earlier findings which suggested that exposure to gamma irradiation caused a certain degree of mutation to occur, thus resulting in an increase in the genetic distance between the irradiated samples and the control, although they were all clonal in origin. However, it also suggested that plants were also capable of undergoing phenotype and DNA repair. The genetic distances between the irradiated samples and the control were shown to be reduced after 4 weeks of recovery.

## Discussion

Numerous mutagenic agents have been used to create and expand the genetic diversity of various species. Among them, gamma radiation (an example of ionizing radiation) has been successfully used in crop improvement and the generation of new cultivars. Due to its shorter wavelength and higher energy, gamma radiation is capable of penetrating deeper into plant tissues^12^, thus inducing a wide range of mutations which may be desirable to farmers or crop breeders, such as disease and pest resistance, semi-dwarfism, early maturity, and improved plant stature^12,13^. Subsequently, the mutants with these desirable traits would be subjected to multi-location field trials (with a minimum of two subsequent generations) to determine their stability, prior to market release^14–16^. For example, the rice cultivar ‘Zhefu 802’ which was produced through gamma-ray induction was widely planted in China in the 1990s, due to its excellent properties (increased tolerance to rice blast^17^). In addition, gamma-induced *Saccharum officinarum* mutants have been reported to show resistance towards the sugarcane mosaic virus, increased growth performance (improved plant height, number of green leaves per plant and higher tiller number), increased drought tolerance as well as producing higher yield and sugar content^18,19^.

Nevertheless, the efficacy of gamma irradiation in inducing desirable mutations is dependent on its irradiation dose. In a reported study by Amjad and Anjum^20^, exposure to higher doses of gamma irradiation led to severe and drastic effects, and relatively lower doses often resulted in changes of plant growth characteristics and genetic variability in the plant genome. According to Amjad and Anjum^20^ in their study on seedling growth of onion (*Allium cepa* L.), gamma irradiation at higher doses was observed to cause a severe reduction in seedling length compared to lower doses. A study by Al-Safadi and Simon^21^ also reported that exposure to higher doses of gamma radiation caused the survival rate of carrots to reduce by about 50% due to delayed germination. It was also reported to be caused by the blockage of the AUX synthesis pathway, DNA synthesis inhibition, and production of growth inhibitors^22^.

The gamma-induced damage in the DNA structures was observed to adversely influence the morphological characteristics of the plants such as the root and shoot lengths^21^. Although plants are immobile, they have developed a unique mechanism termed phenotypic plasticity, to survive their stressful environments. The plant characteristics can be maintained by repairing the damage and the alteration in the genetic composition brought about by the stress factors^23^. According to Goh, et al.^24^, acute and chronic exposure to gamma radiation (at 200 Gy) reduced the height, silique number, and silique length of *Arabidopsis* plants, coupled with a decrease in antioxidant enzyme levels such as catalase (CAT) and peroxidase (POD). Several genes related to ROS signaling such as the genes which encode for NADPH oxidase, zinc finger proteins, heat shock factors, WRKY DNA-binding proteins, and calcium binding proteins were also found to be up-regulated^24^. In addition, Hong, et al.^25^ reported that gamma-irradiated wheat (*Triticum aestivum*) exhibited increased expression of anthocyanin biosynthesis genes such as flavanone 3-hydroxylase (*F3H*), dihydroflavonol reductase (*DFR*), anthocyanin reductase (*ANS*) and UDPG-flavonoid glucosyl transferase (*UFGT*), which helped to modulate the generation of stress-induced ROS brought upon by the exposure to gamma radiation. These observations suggested that irradiation-induced stress prompted plants to activate their antioxidant systems to scavenge ROS, as part of their defense and repair mechanisms. However, the effects of exposure to gamma radiation on plant plasticity and the plant’s DNA damage repair is not yet fully understood. The information on the extent of damage that plants can withstand, and how plants modulate their repair pathways in response to such extensive irregularities are essential in building a foundation of knowledge that aids in developing potential methods for generating more resilient plant cultivars with better yield. This will contribute towards the achievement of one of the sustainable development goals, SDG2 (zero hunger). In the current study, gamma radiation was used as a stress factor to induce DNA damage in plants (clonal *Ananas comosus* var. MD2). Following the exposure to gamma radiation, the height of the irradiated plantlets was observed to be significantly lower than the control (non-irradiated), as the exposure to gamma irradiation at 400 Gy had caused the plantlets to become stunted. However, the plantlets continued to grow as they recover with time. A similar observation has been previously reported in physic nut (*Jatropha curcas* L.), where the exposure to gamma radiation caused stunting^26^. According to Datta^27^, the reduction of plant height could be due to the damage to plant tissues which induced the inhibitory effect on the exposed plants. This observation was also reported in rice seedlings by El-Aishy, et al.^28^. The rice seedlings exhibited an increased number of stunted roots after being exposed to high doses of gamma radiation, due to alterations in its genome^28^. Analysis of physiological data in the current study further supported these observations. We found that photosynthetic parameters such as A, E and gs of the irradiated plantlets were impaired due to exposure to gamma radiation (Fig. 3). A similar observation was recorded by Singh, et al.^29^ where the photosynthetic rate, transpiration rate (E), and stomatal conductance (gs) of wheat was significantly inhibited when the plants were exposed to a high dose of gamma radiation (> 100 Gy). This is likely due to poor nitrogen and carbon assimilation efficiency. However, the irradiated plantlets in the current study were observed to slowly recover post-irradiation, as shown by the increase in the PI, A, and WUE after 2 weeks.

According to Vijayan^30^, ISSR is one of the quickest marker detection system with high reproducibility. It could detect more polymorphism than other detection methods such as mtDNA, cpDNA, and RAPD in closely related plant groups. ISSR primers could be used to detect misses in sequence repeats, deletion or insertion in the genome which can modify the distance between the repeats, all of which result in polymorphisms of DNA fragments. In a previous study by Huang, et al.^31^, ISSR markers were successfully used to determine the genetic stability of *Platanus acerifolia*. In the study, 86 out of 103 generated bands were found to be polymorphic. These findings further reaffirmed the capability of ISSR markers to efficiently detect polymorphism compared to other markers, thus they are suitable to be used in detecting plant genetic variability^31^. Apart from that, the use of ISSR primers in genetic diversity assessments had also been reported in many plant species such as *Ananas comosus* (L.) Merr^32,33^ and mulberry^34^.

In addition, ISSR markers can also be used to locate and isolate mutations for the purpose of genetic discrimination^35^. In a study conducted by Rathore, et al.^36^, ISSR markers were found to be a reliable method that enabled a rapid evaluation of the occurrence of somaclonal variation between samples. Moreover, ISSR markers have also been successfully used to reveal somaclonal variation in yacon *(Smallanthus sonchifolius)*^11^. In the current study, the clonal nature of the *A. comosus* plantlets was also initially confirmed through ISSR analysis (Supplementary Fig. S1, S2). The same ISSR markers were subsequently used to determine the genetic variation between irradiated and control (non-irradiated) plantlets at various time points, post-exposure to gamma radiation. Data analysis revealed that the highest genetic distance (0.2073) was observed between the non-irradiated (control) and gamma-irradiated plantlets after 1 day to 2 weeks post-irradiation (Table 4). However, the genetic distance between the control and irradiated plantlets was observed to decrease after 4 weeks of recovery, resulting in a genetic distance of 0.1017. The UPGMA dendrogram also revealed similar outcomes, which further supports earlier findings where the exposure to gamma irradiation caused mutations to occur, resulting in an increase of genetic distance between the irradiated samples and the control, although they were all clonal in origin. Parallel to these findings, a study conducted by Al-Safadi and Simon^21^ also showed the presence of chromosomal abnormalities among the carrot (*Daucus carota* L.) cultures after being exposed to gamma radiation.

However, plants are also capable of undergoing phenotype and DNA repair, as indicated by the outcomes of this study, where the genetic distances between the irradiated *A. comosus* samples and the control were shown to be reduced after 4 weeks of recovery. Plants have been reported to be able to repair DNA damages in their genome by employing various DNA damage repair (DDR) mechanisms such as photoreactivation, nucleotide excision repair (NER), base excision repair (BER) and mismatch repair (MMR)^37^. In response to exposure to ionizing radiation, BER will be employed to repair damaged or modified bases in the DNA, while NER functions to remove any lesions in the DNA structure. However, a more radical approach is often employed to repair single or double strand breaks such as through the break repair pathways^38,39^. The break repair pathways including homologous recombination (HR) and non-homologous end-joining (NHEJ) are essential in plants for the preservation of their genetic stability^40,41^. Similar to the outcomes of this study, it has been reported that the DNA damages that occurred in the tobacco seedlings after the exposure to gamma radiation had been repaired by DDRs after 4 weeks of recovery^42^. This observation was further ascertained by the morphological observation of the tobacco seedlings, which found that the damage in the leaf samples was reduced after the recovery period^42^.

## Conclusion

Exposure to gamma radiation was found to cause DNA damage in irradiated samples. This resulted in higher genetic variation in irradiated samples compared to control. However, evidence of phenotype and DNA repair following the exposure to gamma irradiation was also observed. The genetic distance between the irradiated and control plantlets was observed to decrease after 4 weeks of recovery. This was further strengthened by the morphological and physiological observations of the samples, which showed that the irradiated plantlets continued to grow during the recovery period, although they exhibited a lower performance index than the control. Our findings also demonstrated that ISSR can serve as an efficient marker system to assess genetic variation in the plant genome, with high reproducibility and sensitivity.

## Supplementary Information

Supplementary Information.

## Data Availability

The datasets used and/or analysed during the current study are available from the corresponding author on reasonable request.
