# Supplementary material for: Genetic and phenotype recovery of Ananas comosus var. MD2 in response to ionizing radiation
Source: Sci Rep. 2023 Jan 5;13:182. doi: 10.1038/s41598-022-26745-3 (PMC9814699; doi:10.1038/s41598-022-26745-3)
Supplement: Supplementary file 1 — Supplementary Information. [file 41598_2022_26745_MOESM1_ESM.pdf]

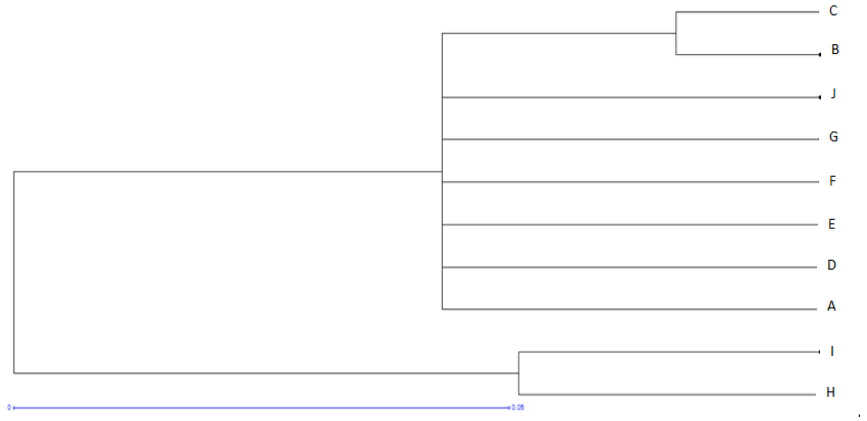

Fig. S1. UPGMA dendrogram generated from 10 randomly selected clonal plantlets. A-J: Plantlets A-J

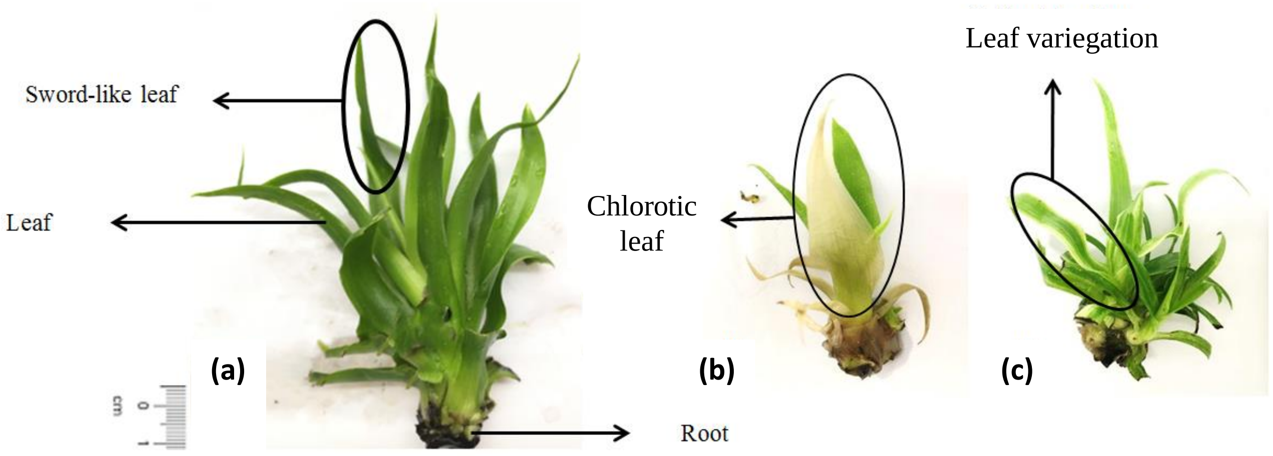

Fig. S2. (a) Morphology of the non-irradiated (control) *Ananas comosus* var. MD2 plantlet, (b) gamma-irradiated plantlet after 2 weeks of recovery period showing leaf chlorosis and (c) gamma-irradiated plantlet after 2 weeks of recovery period showing leaf variegation.

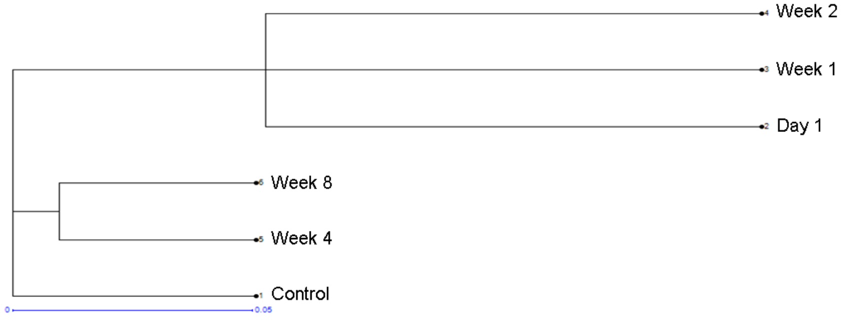

Fig. S3. UPGMA dendrogram generated from 10 randomly selected irradiated and non-irradiated (control) clonal plantlets at various post-recovery periods. Control: control plantlets were not irradiated; Day 1, Week 1, Week 2, Week 4 and Week 8: recovery period after gamma irradiation respectively.

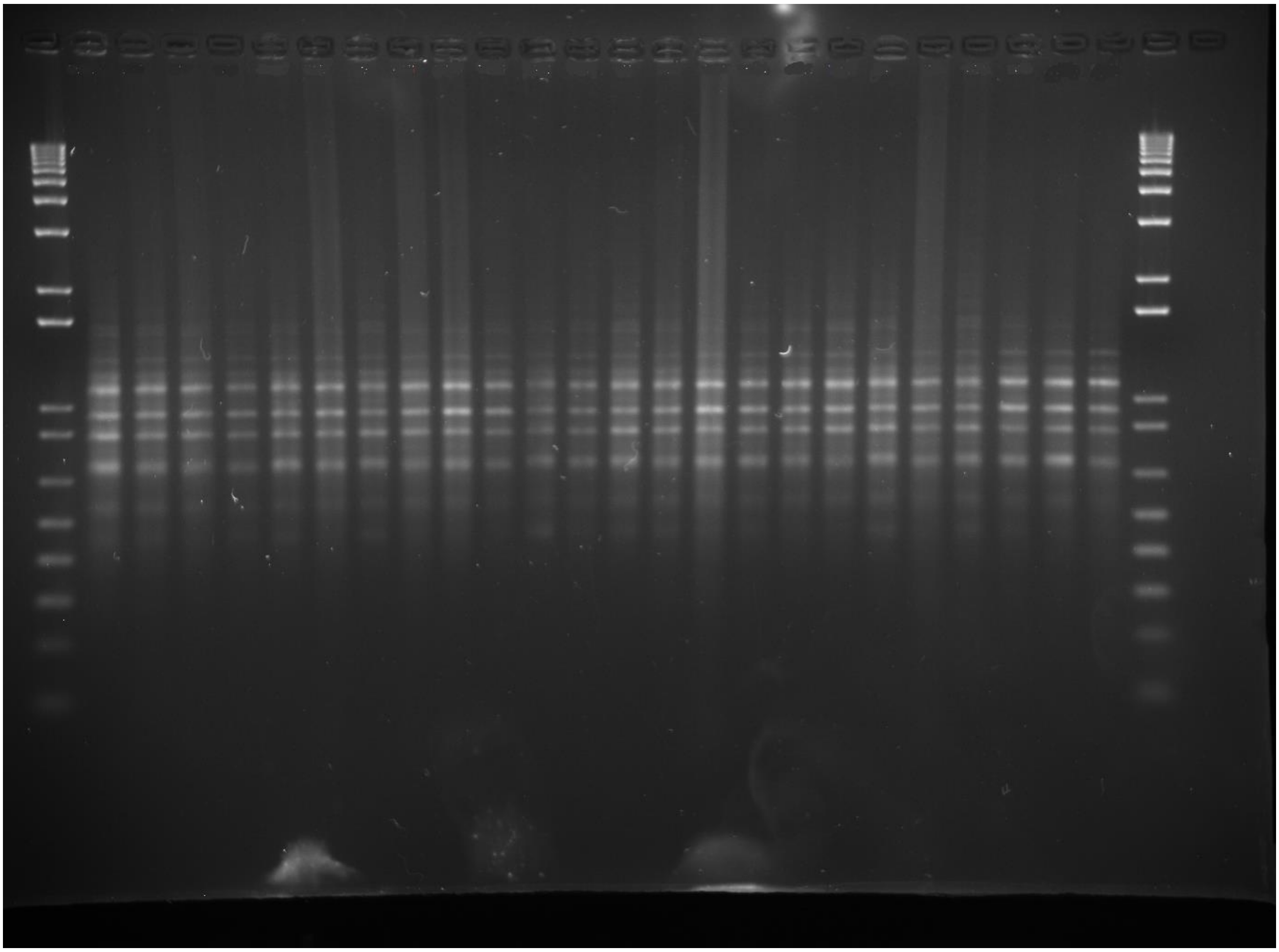

Fig. S4. The original gel image obtained using ISSR primer UBC 840.

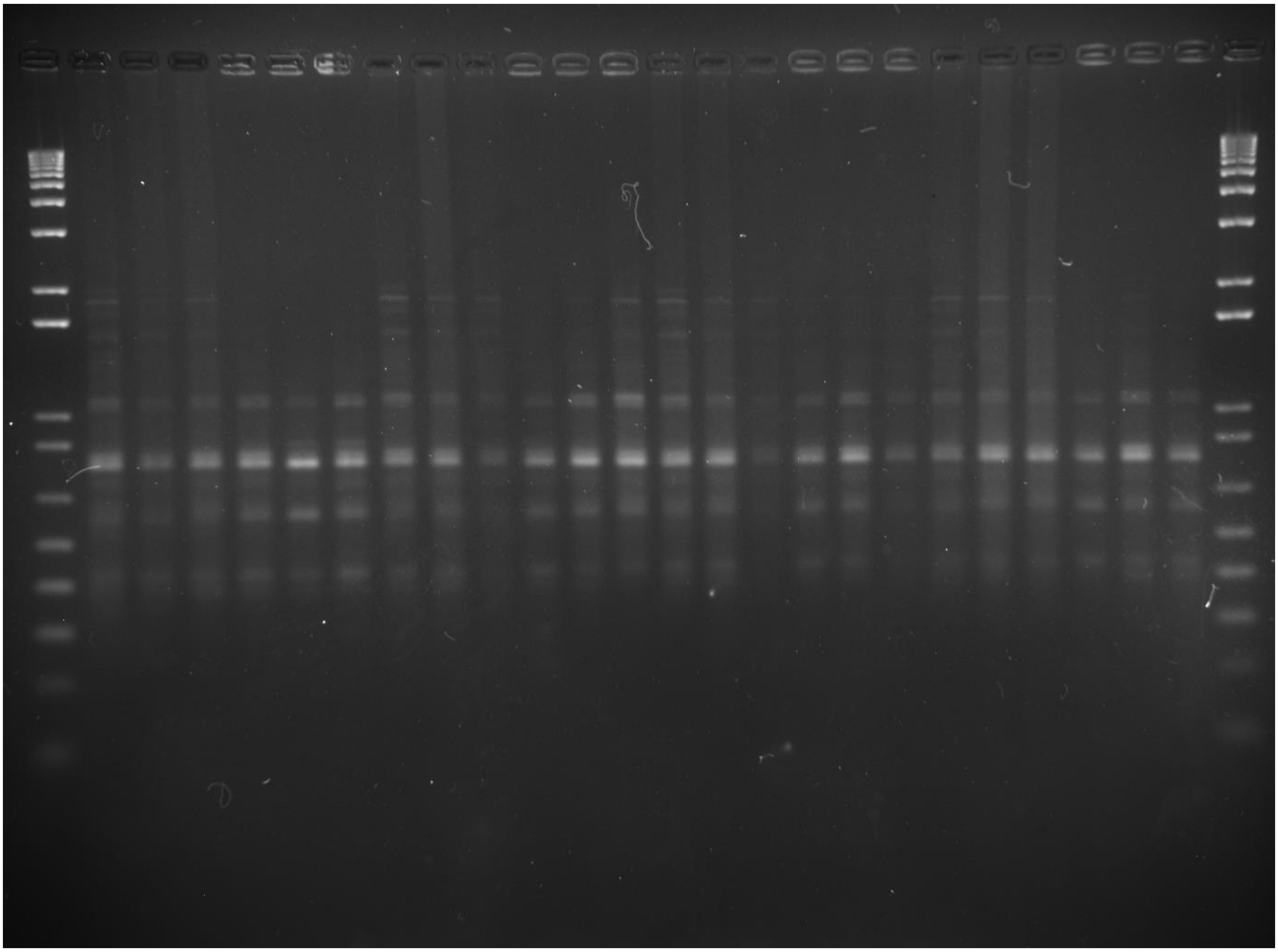

Fig. S5. The original gel image obtained using ISSR primer UBC 841.

Table S1. List of ISSR primers used in the analysis [11].

Primers code (UBC)	Sequence 5'-3'	Annealing temperature (°C)
UBC 807	$(AG)_8T$	46.5
UBC 809	$(AG)_8G$	48.0
UBC 829	$(TG)_8C$	52.5
UBC 834	$(AG)_8YT$	52.0
UBC 840	$(GA)_8YT$	46.5
UBC 841	$(GA)_8YC$	52.0
UBC 855	$(AC)_8YT$	53.0
UBC 856	$(AC)_8YA$	54.0
